# Causal Relationship Between Epilepsy, Status Epilepticus and Sleep-Related Traits: A Bidirectional Mendelian Randomization Study

**DOI:** 10.3390/brainsci15070749

**Published:** 2025-07-14

**Authors:** Yong-Won Shin, Sang Bin Hong

**Affiliations:** 1 Department of Critical Care Medicine, Seoul National University Hospital, Seoul 03080, Republic of Korea; 2 Center for Hospital Medicine, Department of Neurology, Seoul National University Hospital, Seoul 03080, Republic of Korea; hong.sangbin@gmail.com

**Keywords:** epilepsy, status epilepticus, sleep-related traits, Mendelian randomization

## Abstract

**Background/Objectives**: Epilepsy and sleep disturbances frequently co-occur, yet the causal nature of this relationship remains uncertain, particularly in relation to epilepsy subtypes and status epilepticus. We investigated potential bidirectional causal associations between sleep-related traits and epilepsy, including subtypes and status epilepticus, using Mendelian randomization (MR). **Methods**: We conducted two-sample MR using genome-wide association study (GWAS) summary statistics from European ancestry cohorts. Epilepsy, its subtypes, and status epilepticus were analyzed using data from the International League Against Epilepsy Consortium on Complex Epilepsies (ILAE) and the FinnGen study. Nine self-reported sleep-related traits were derived from the UK Biobank-based GWAS. Causal estimates were primarily obtained using inverse variance weighted models with additional MR analysis methods. Pleiotropy and heterogeneity were assessed to enhance the robustness of the finding. **Results**: Several subtype-specific associations were identified, with direction and statistical significance varying across cohorts and subtypes. After correction for multiple testing and filtering for tests with ≥10 instrumental variables to ensure robust and reliable MR estimates, several consistent and potentially mutually reinforcing associations emerged. In the ILAE cohort, focal epilepsy with hippocampal sclerosis was associated with an increased risk of insomnia, and juvenile myoclonic epilepsy with reduced sleep duration. In the FinnGen cohort, overall epilepsy was associated with increased risk of both insomnia and daytime sleepiness. In reverse MR, daytime sleepiness and napping were associated with increased risk of epilepsy, while daytime napping and frequent insomnia symptoms were linked to elevated risk of status epilepticus. **Conclusions**: Our findings reveal subtype-specific and bidirectional causal links between epilepsy and sleep-related traits. These results highlight the biological interplay between epileptic networks and sleep regulation and underscore the need for further clinical and mechanistic studies.

## 1. Introduction

Epilepsy is a neurological disorder characterized by recurrent seizures, with a diverse and complex etiology, and is frequently associated with a range of comorbidities and complications, including cognitive impairment, psychiatric disorders, and sleep disturbances [1,2]. Underlying etiologies include genetic predisposition, structural brain abnormalities, metabolic disorders, infections, and autoimmune mechanisms [3].

Sleep and circadian rhythm disturbances, in particular, have been shown to be closely linked with epilepsy. Previous studies have reported that patients with epilepsy experience higher rates of sleep disturbances, including insomnia, excessive daytime sleepiness, and other sleep-related symptoms, as well as alterations in sleep architecture and quality [2,4,5]. A comprehensive review reported that insomnia affects approximately 36% to 74% of adults with epilepsy, and remains between 29% and 51% when restricted to moderate-to-severe symptoms (Insomnia Severity Index ≥15) [4]. Previous reports have suggested a higher prevalence of excessive daytime sleepiness in epilepsy [6,7], with the prevalence estimates ranging from 10% to 47.5% [8]. However, a recent meta-analysis found no significant association between epilepsy and excessive daytime sleepiness when assessed using the Epworth Sleepiness Scale, although it still highlighted consistently poorer overall sleep quality in patients with epilepsy compared to controls [9]. In a study involving 200 patients with epilepsy (100 generalized and 100 focal), 41% reported increased daytime sleepiness following nocturnal seizures; 16% reported needing an immediate nap after a diurnal generalized seizure, and 5% experienced disrupted nocturnal sleep after such seizures [10]. The same study also reported that 45% of patients with generalized epilepsy identified sleep deprivation as a seizure trigger, significantly higher than the 24% observed in patients with focal epilepsy [10]. Additionally, an EEG-based longitudinal study demonstrated that day-to-day variations in sleep duration were associated with corresponding changes in seizure probability [11]. Sleep deprivation is known to increase cortical excitability and facilitate epileptiform discharges, serving as a potential trigger for seizures [12,13]. Notably, some patients exhibit pure sleep-related epilepsy, where all seizures are confined to sleep periods, highlighting the strong influence of sleep on seizure expression [14]. In addition, investigation into chronotypes among adult patients with epilepsy has revealed an overall tendency toward morningness. Some studies have reported that adult patients with generalized epilepsy more frequently exhibit eveningness chronotypes compared to those with focal epilepsy or healthy controls [15].

Sleep-related hypermotor epilepsy (SHE) is a form of focal epilepsy characterized by brief, stereotyped motor, most commonly hypermotor, seizures that occur predominantly during sleep [16]. SHE was previously referred to as nocturnal frontal lobe epilepsy; however, the terminology has changed as SHE can originate outside the frontal lobe and, more importantly, because its defining feature is its occurrence during sleep, rather than its timing within the day. SHE exemplifies a subtype of epilepsy in which sleep plays a strong causal role. Another representative subtype is juvenile myoclonic epilepsy (JME), a common form of generalized epilepsy characterized by myoclonic and generalized tonic–clonic seizures [17]. Myoclonic seizures in JME typically occur within the first hour after awakening and are particularly sensitive to sleep deprivation. In general, tonic, tonic–clonic, and hypermotor seizures tend to be more frequently observed during sleep, while other types of seizures are more commonly observed during wakefulness [18,19]. Longitudinal monitoring has also revealed clear circadian and multidien rhythms in seizure occurrence [20,21]. These findings support the notion that sleep and circadian rhythm are causally linked to epilepsy, with patterns of association that may differ across epilepsy subtypes. Such manifestations may reflect both the downstream effects of epilepsy and innate, genetically driven patterns of sleep and circadian regulation that predispose to the development or expression of epilepsy.

Mendelian randomization (MR) is a statistical method used to infer causal relationships between exposures and outcomes, commonly by using germline genetic variants as instrumental variables (IVs). It is particularly valuable for addressing limitations of observational epidemiologic studies, such as confounding and reverse causation [22]. Recent MR studies have begun to uncover causal links between sleep-related traits and epilepsy. One study reported genetically inferred associations between insomnia or evening chronotype and increased epilepsy risk, and also found that epilepsy may, in turn, influence sleep duration [23]. However, these analyses treated epilepsy as a single entity and assessed only a limited number of sleep-related traits. Other studies, using phenome-wide or cross-population MR approaches to assess a broader range of sleep-related traits and evaluate epilepsy as a whole or at the level of major subtypes, such as focal and generalized epilepsy, have yielded inconsistent or null findings, partly due to limited trait resolution and unidirectional focus [24,25]. Although not specifically focused on the relationship between epilepsy and sleep-related traits, additional MR studies support a broader biological connection between epilepsy, sleep, and psychiatric traits. For instance, MR evidence suggests that genetic liability to attention-deficit/hyperactivity disorder increases the risk of generalized epilepsy, while focal epilepsy increases the risk of obsessive–compulsive disorder [26]. Moreover, recent MR studies have identified bidirectional associations between sleep-related traits and brain structural changes, suggesting neuroanatomical pathways that may also underlie epilepsy [27]. These findings, summarized in Table S1, underscore the need for subtype-specific and bidirectional MR analyses to better delineate the causal relationships between epilepsy and sleep-related traits.

In addition to epilepsy subtypes, status epilepticus represents a clinically distinct and severe seizure condition that is frequently associated with drug-resistant epilepsy, multiple complications, and poor outcomes. Despite its clinical significance, there is a lack of MR-based studies addressing status epilepticus and its relationship with sleep-related traits.

Building on prior evidence of bidirectional associations between sleep and epilepsy, we hypothesized that multiple epilepsy subtypes and status epilepticus are causally associated with sleep-related traits, and vice versa, with potential subtype-specific differences in the direction and strength of these relationships. To test this hypothesis, we employed MR analysis using large-scale, well-powered genome-wide association study (GWAS) datasets from national biobanks (UK Biobank, FinnGen) and an international consortium, the International League Against Epilepsy Consortium on Complex Epilepsies (ILAE), all of which have been widely used in genetic studies of neurological and behavioral traits. We selected sleep-related traits and epilepsy phenotypes based on prior epidemiologic findings and data availability, and implemented a rigorous MR protocol that includes robust instrument selection, pleiotropy filtering, and multiple sensitivity analyses to validate causal inference. This study aims to refine our understanding of potential causal associations between sleep-related traits and a broad spectrum of epilepsy phenotypes, including both epilepsy subtypes and status epilepticus, which have been underexplored in previous MR research.

## 2. Materials and Methods

### 2.1. Study Design

**Figure 1:**
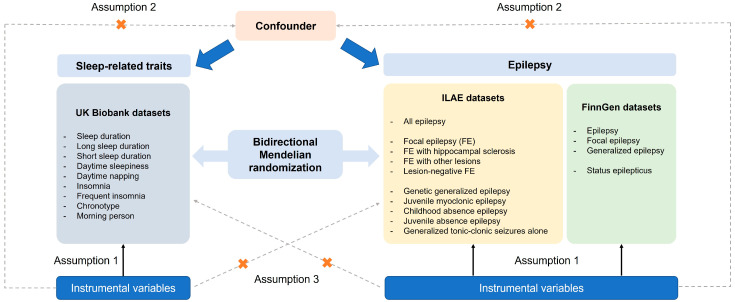
An overview of the study design including three core assumptions.

We performed two-sample MR analysis to evaluate the potential causal relationships between epilepsy, status epilepticus, and sleep-related traits. The analysis was conducted using single-nucleotide polymorphisms (SNPs) as IVs. MR relies on three core assumptions: (1) The selected IVs are strongly associated with the exposure (relevance); (2) the association between the IVs and the outcome is independent of confounding factors (independence); (3) the IVs affect the outcome only through the exposure (exclusion restriction) [28]. Instrument selection and MR analysis were based on publicly available GWAS summary statistics derived from individuals of European ancestry, in order to minimize potential bias due to population stratification. Causal relationships were evaluated bidirectionally, with sleep traits tested as both exposures and outcomes relative to epilepsy and status epilepticus. Figure 1 illustrates the design of the current study. The current study was not prospectively registered.

### 2.2. Data Sources

The current study included nine self-reported sleep-related traits: sleep duration, long sleep duration (≥9 h), short sleep duration (≤6 h), daytime sleepiness, daytime napping, insomnia, frequent insomnia symptoms, chronotype, and morning person. Summary-level GWAS data for these traits were obtained from previously published studies utilizing the UK biobank cohort [29,30,31,32,33,34]. For sleep duration, participants were asked “About how many hours sleep do you get in every 24 h? (please include naps)”, with answers recorded in hour increments [29]. Responses of less than 3 h or more than 18 h were excluded from analysis. For daytime sleepiness, participants were asked “How likely are you to dose off or fall asleep during the daytime when you don’t mean to? (e.g., when working, reading or driving)”. Responses were scored on a four-point scale: 1 = “Never/rarely”, 2 = “Sometimes”, 3 = “Often”, and 4 = “All of the time” [30]. Responses of “Do not know” and “Prefer not to answer” were treated as missing. For daytime napping, participants were asked “Do you have a nap during the day?”, with responses “Never/rarely”, “Sometimes”, and “Usually” were treated as a continuous variable in the GWAS [31]. “Prefer not to answer” responses were excluded. For insomnia, participants were asked “Do you have trouble falling asleep at night or do you wake up in the middle of the night?”, with response options “Never/rarely”, “Sometimes”, “Usually”, and “Prefer not to answer”. Two dichotomous definitions were used: insomnia (“sometimes/usually” vs. “never/rarely”) and frequent insomnia symptoms (“usually” vs. “sometimes/never/rarely”). “Prefer not to answer” responses were set to missing. GWAS summary statistics for each definition were obtained from separate studies, as the classification criteria differed between the two sources [32,33]. For chronotype, participants were asked “Do you consider yourself to be?”, with responses “Definitely a ‘morning’ person”, “More a ‘morning’ than ‘evening’ person”, “More an ‘evening’ than a ‘morning’ person”, “Definitely an ‘evening’ person”, “Do not know” or “Prefer not to answer”, which were coded as 2, 1, −1, −2, 0 and missing, respectively [34]. For the morning person phenotype, individuals who responded “Definitely a ‘morning’ person” or “More a ‘morning’ than ‘evening’ person” were classified as morning person, while those who responded “More an ‘evening’ than a ‘morning’ person” or “Definitely an ‘evening’ person” were classified as control (evening person); “Do not know” or “Prefer not to answer” were excluded. Further details regarding the data collection and GWAS analysis for each trait can be found in the original publications [29,30,31,32,33,34].

For epilepsy and status epilepticus, we used two GWAS summary statistic sources: ILAE [35] and the FinnGen study [36]. From the ILAE dataset, which includes a multi-ancestry GWAS meta-analysis, we used the summary statistics restricted to individuals of European ancestry. The dataset includes GWAS results for all epilepsy cases as well as two broad subgroups: focal epilepsy (FE) and genetic generalized epilepsy (GGE). Within each of these, several clinically relevant subtypes were analyzed. For FE, subtypes included FE with hippocampal sclerosis (HS), FE with other lesions, and lesion-negative FE. For GGE, subtypes included JME, childhood absence epilepsy (CAE), juvenile absence epilepsy (JAE), and generalized tonic–clonic seizures alone (GTCSA). All subtypes were analyzed using a common control population. GWAS summary statistics for unclassified epilepsy or for unclassified subtypes within FE and GGE were not publicly available and thus were not included in this study.

To assess the robustness of our findings and to extend MR analysis to status epilepticus, we additionally used the R12 release of the FinnGen dataset, a large-scale Finnish biobank initiative that includes genetic and health data from over 500,000 individuals [36]. The following four phenotype datasets from FinnGen were used in the analysis: epilepsy, focal epilepsy, generalized epilepsy, and status epilepticus.

A summary of the GWAS data sources used in this study is provided in Table 1.

### 2.3. Instrumental Variable Selection

To fulfill the core assumptions of MR, we first selected SNPs reaching genome-wide significance (*p* < 5 × 10^−8^). For epilepsy-related traits, a relaxed threshold of *p* < 5 × 10^−6^ was used due to the limited number of genome-wide significant SNPs. Subsequently, linkage disequilibrium (LD) clumping was performed using an *r*^2^ threshold of <0.001 within a 10 Mb window, based on the European reference panel from the 1000 Genomes Project. SNPs with allele mismatches between exposure and outcome, a minor allele frequency <0.01, or palindromic SNPs were removed. Only SNPs with F statistics >10 were retained to reduce the impact of weak instrument bias. To avoid horizontal pleiotropy, SNPs that showed genome-wide significant associations (*p* < 5 × 10^−8^) with both the exposure and outcome traits were excluded. In addition, we screened the remaining SNPs in the GWAS Atlas (https://atlas.ctglab.nl/, accessed on 24 May 2025), and variants significantly associated with potential confounders, including schizophrenia, attention-deficit/hyperactivity disorder, depression, neuroticism, and body mass index, were also removed. We also performed a leave-one-out analysis to identify influential SNPs whose removal substantially altered the MR estimates. Finally, we applied the MR-PRESSO (Mendelian Randomization Pleiotropy RESidual Sum and Outlier) method [37] to detect and remove outlier SNPs that may introduce horizontal pleiotropy.

### 2.4. Statistical Analysis

Bidirectional MR analysis was performed using a multiplicative random-effects inverse variance weighted (IVW) estimate as the primary method. Analyses using MR-Egger, weighted median, simple mode, and weighted mode methods were additionally conducted to test the robustness of the causal effect. The Wald ratio was used to obtain an estimate from each SNP. Heterogeneity among SNP-specific estimates was assessed using Cochran’s Q statistic and the *I*^2^ statistic under the IVW model. Evidence of directional horizontal pleiotropy was assessed using the MR-Egger intercept. All analyses were conducted using R software packages, including TwoSampleMR (v0.6.15) and MRPRESSO (v1.0). Multiple testing correction was applied using the Benjamini-Hochberg method across multiple exposures for each outcome. A *p* value < 0.05 was considered statistically significant. Representative R code and parameter settings used in the MR analysis are provided in the Supplementary Materials.

## 3. Results

### 3.1. Causal Effects of Epilepsy on Sleep-Related Traits

**Figure 2:**
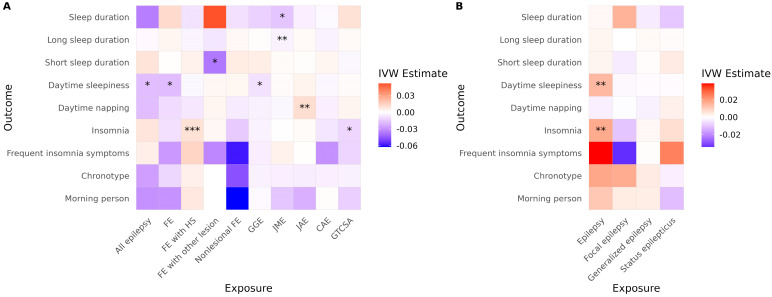
Causal effects of epilepsy on sleep-related traits across cohorts. Heatmaps show IVW estimates for each exposure-outcome pair in (**A**) the ILAE and (**B**) the FinnGen cohorts. Color intensity represents the magnitude and direction of the IVW estimate (red = positive; blue = negative). Asterisks indicate the level of supporting evidence from additional MR methods (* *p* < 0.05 by IVW only; ** supported by one additional method; *** supported by ≥2 methods among MR-Egger, weighted median, simple mode, or weighted mode). Significance was determined using nominal *p* values. Only associations with at least two instrumental variables enabling IVW analysis are shown. Abbreviations: IVW, inverse variance weighted; FE, focal epilepsy; HS, hippocampal sclerosis; GGE, genetic generalized epilepsy; JME, juvenile myoclonic epilepsy; CAE, childhood absence epilepsy; GTCSA, generalized tonic–clonic seizures alone.

In the ILAE cohort, several significant associations were observed using the IVW method (Figure 2A, Table 2). FE with HS was associated with an increased risk of insomnia, and JME was associated with reduced sleep duration and a higher risk of short sleep duration. JAE was associated with an increased risk of daytime napping. Conversely, all epilepsy, FE, and GGE were significantly associated with reduced daytime sleepiness. FE with other lesions was associated with a reduced risk of short sleep duration. GTCSA was associated with a decreased risk of insomnia. In the FinnGen cohort, overall epilepsy was associated with an increased risk of both insomnia and daytime sleepiness (Figure 2B, Table 2). However, none of the generalized epilepsy, FE, or status epilepticus phenotypes showed a significant causal effect on any sleep-related traits.

After correcting for multiple comparisons, JME remained significantly associated with reduced sleep duration (*p* = 0.040), and FE with Other lesions remained negatively associated with short sleep duration (*p* = 0.048). The association between FE with HS and insomnia did not reach conventional significance (*p* = 0.060), but became significant (*p* = 0.042) when multiple testing correction was applied only within epilepsy subtypes (excluding all epilepsy, FE, and GGE). All other associations lost statistical significance after multiple testing correction. In the FinnGen cohort, epilepsy remained significantly associated with both insomnia (*p* = 0.014) and daytime sleepiness (*p* = 0.0009) following correction.

No evidence of directional horizontal pleiotropy was found in any of the significant associations (all Egger intercept *p* > 0.05). Cochran’s *Q* test indicated low to moderate heterogeneity across SNP-specific estimates for three associations, with *I*^2^ values ranging from 24% (FE to daytime sleepiness) to 53% (GGE to daytime sleepiness), as shown in Table 2. It is noteworthy that daytime sleepiness was affected in opposite directions in the two cohorts. Additionally, caution is warranted when interpreting results involving GGE and FE to daytime sleepiness due to observed heterogeneity. Daytime sleepiness was differently affected in the two cohorts. Results for FE with other lesions to short sleep duration and GTCSA to insomnia also require caution, as only two and three SNPs, respectively, were available as instrumental variables for these analyses. Full MR results are presented in Table S2.

### 3.2. Causal Effects of Sleep-Related Traits on Epilepsy

**Figure 3:**
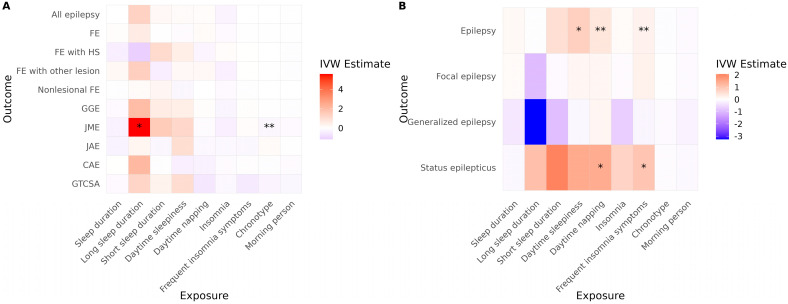
Causal effects of sleep-related traits on epilepsy and status epilepticus. Heatmaps show IVW estimates for each exposure-outcome pair in (**A**) the ILAE and (**B**) the FinnGen cohorts. Color intensity represents the magnitude and direction of the IVW estimate (red = positive; blue = negative). Asterisks indicate the level of supporting evidence from additional MR methods (* *p* < 0.05 by IVW only; ** supported by one additional method). Significance was determined using nominal *p* values. Only associations with at least two instrumental variables enabling IVW analysis are shown. Abbreviations: IVW, inverse variance weighted; FE, focal epilepsy; HS, hippocampal sclerosis; GGE, genetic generalized epilepsy; JME, juvenile myoclonic epilepsy; CAE, childhood absence epilepsy; GTCSA, generalized tonic–clonic seizures alone.

In the ILAE cohort, long sleep duration was associated with an increased risk of JME (Figure 3A, Table 3). A more evening-type chronotype was also associated with an elevated risk of JME. No other sleep-related traits demonstrated significant causal associations with epilepsy risk in this cohort. In the FinnGen cohort, daytime sleepiness, daytime napping, and frequent insomnia symptoms were significantly associated with an increased risk of JME (Figure 3B, Table 3). Additionally, both daytime napping and frequent insomnia symptoms showed significant associations with an increased risk of status epilepticus.

After correction for multiple comparisons, none of the associations observed in the ILAE cohort remained statistically significant. However, in the FinnGen cohort, daytime sleepiness and daytime napping continued to show significant associations with JME (*p* = 0.039 for both). Furthermore, the associations of daytime napping and frequent insomnia symptoms with status epilepticus remained significant (*p* = 0.028 for both). No evidence of directional horizontal pleiotropy or significant heterogeneity was detected for any of the significant associations in the reverse MR analysis. Full reverse MR results are presented in Table S2.

## 4. Discussion

In this two-sample MR analysis, we found that epilepsy and its subtypes exhibited distinct causal relationships with several sleep-related traits. Conversely, several sleep traits also showed evidence of causal effects on epilepsy, status epilepticus and, potentially, JME. The direction and magnitude of these associations varied across epilepsy subtypes and between cohorts. While most significant associations demonstrated consistent patterns across datasets, the direction of effect for daytime sleepiness differed by cohort. After adjusting for multiple comparisons, the majority of significant findings supported a bidirectional negative association between epilepsy and sleep-related traits. FE with other lesions was associated with a decreased risk of short sleep duration, whereas other epilepsy subtypes and sleep-related traits, such as overall epilepsy, FE with HS, JME, insomnia, sleep duration, daytime sleepiness, and daytime napping, showed mutually reinforcing associations, suggesting that impaired sleep and epileptic activity may perpetuate each other. Regarding status epilepticus, both daytime napping and frequent insomnia symptoms were identified as potential causal risk factors, underscoring the relevance of sleep disturbance in severe seizure presentations. Although many associations did not reach statistical significance after correction, and some showed moderate heterogeneity, the overall findings from this bidirectional MR analysis suggest that epilepsy and sleep-related traits can exert mutual adverse influences on one another.

The heterogeneous associations observed between specific epilepsy subtypes and sleep-related traits likely reflect differences in underlying neurophysiology, seizure timing, and sleep–wake regulation mechanisms. Our MR results also indicate that the direction and strength of genetic liability between sleep-related traits and epilepsy subtypes differ, suggesting biologically distinct pathways. Such heterogeneity has also been observed in studies using individual-level clinical data. For example, a recent meta-analysis reported that epilepsy is associated with poor subjective sleep quality, as measured by the Pittsburgh Sleep Quality Index, but not with excessive daytime sleepiness, as assessed by the Epworth Sleepiness Scale [9]. Another study reported a significantly higher prevalence of both short and long sleep duration in adults with epilepsy than in controls [38]. This reinforces the notion that distinct sleep phenotypes may show divergent associations with epilepsy. The presence of such heterogeneity underscores the need for future studies incorporating deeply phenotyped clinical cohorts and mechanistic investigations. We observed several associations that remained significant after multiple testing correction and included a sufficient number of SNPs, supporting the subtype- and trait-specific effects. However, findings from subtypes with fewer SNPs (<10) should still be interpreted with caution.

In this study, we used two separate GWAS summary statistics datasets for epilepsy, which allow for the assessment of consistency across cohorts. Despite some heterogeneity, the bidirectional relationship between epilepsy and insomnia showed a relatively consistent pattern, although differences were observed between the ILAE and FinnGen cohorts. Other sleep-related traits tended to align with the direction of effect observed for insomnia and reduced sleep quality in association with epilepsy. Differences in findings between the two cohorts may, in part, be attributable to their underlying populations. The FinnGen dataset is derived from the Finnish population, whereas the ILAE dataset represents a meta-analysis of multiple European populations. Although the ILAE dataset includes a larger total number of epilepsy cases than FinnGen, the known phenotypic and genetic heterogeneity of epilepsy may offset this advantage in statistical power. Additionally, the distribution of epilepsy subtypes differed between cohorts. In the ILAE dataset (excluding unclassified epilepsy), FE accounted for 68% of the classified cases (14,939/21,891), whereas in the FinnGen cohort, FE comprised 85% (9275/10,965) of classified cases. Given our findings of subtype-specific causal relationships between epilepsy and sleep-related traits, these differences in subtype composition likely contributed to discrepancies in observed associations between cohorts.

An intriguing contextual factor is the high prevalence of insomnia and circadian rhythm disorders in the Finnish population. A prior study reported that the diagnosis of insomnia in Finland is 1.5 to 2 times higher than in other European countries such as France, the UK, Germany, and Italy [39]. The same study found that circadian rhythm disorders were at least twice as prevalent in Finland [39]. This may be influenced by Finland’s significant seasonal variation in daylight exposure due to its northern location, which can disrupt circadian regulation and melatonin production, thereby contributing to sleep disturbances and insomnia [40]. These population-specific environmental and physiological characteristics may explain why associations between sleep-related traits and epilepsy appeared more prominent in the FinnGen cohort compared to the broader European ILAE dataset. Although chronotype was not significantly associated with epilepsy in the FinnGen cohort, further studies incorporating environmental context may help elucidate additional pathways linking sleep, circadian biology, and epilepsy.

Among the various associations identified in this study, one particularly intriguing finding is the increased risk of insomnia specifically associated with FE with HS. This was the only epilepsy subtype in the ILAE cohort to show a statistically significant causal relationship with insomnia. This observation may be biologically grounded in the unique vulnerability of the subiculum, the primary output region of the hippocampal formation, which plays a critical role in sleep–wake regulation [41]. The ventral subiculum, in particular, projects extensively to multiple brain regions involved in sleep–wake control, including arousal-promoting, sleep-promoting, circadian, and neuromodulatory nuclei [41]. Hippocampal sclerosis-associated subicular damage may contribute causally to insomnia by impairing communication with multiple sleep-regulating centers. Supporting this, animal model studies have shown that manipulation of inhibitory interneurons in the subiculum alters circadian seizure timing [42].

There is a case report describing disrupted sleep structure characterized by a reduction in both slow-wave and rapid eye movement sleep for four consecutive nights following an episode of generalized status epilepticus [43]. Encephalopathy related to status epilepticus during slow sleep (ESES), also referred to as continuous spike and wave during sleep (CSWS), further illustrates the pathological interplay between sleep and status epilepticus. ESES is a rare childhood epilepsy syndrome characterized by neurologic deterioration of cognitive, behavioral and/or motor domains along with EEG findings of diffuse spike-and-wave discharges occupying the majority of slow-wave sleep [44]. Over 20% of developmental and/or epileptic encephalopathy cases associated with ESES were attributed to monogenic variants in a study, suggesting a genetic influence on both status epilepticus and sleep regulation [45]. Although sleep-related traits, including insomnia and epilepsy, have been studied in epilepsy, their specific relationship with status epilepticus remains underexplored. Given that status epilepticus is a distinct clinical entity rather than merely a subtype or complication of epilepsy, dedicated studies are needed to investigate its relationship with sleep-related traits as an independent phenotype.

There are several considerations in interpreting the findings of this study. First, although various epilepsy subtypes were evaluated in relation to sleep-related traits, the majority of comparisons were either non-significant or significant in only one cohort. This may reflect weak or absent associations, but the relatively small sample sizes for some subtypes may have limited the power to detect true causal relationships. The substantial etiological heterogeneity within epilepsy itself also complicates interpretation and may contribute to discrepancies in findings between cohorts. Second, limitations related to diagnostic accuracy and subtype classification should also be considered. The FinnGen dataset is based on electronic health records, with epilepsy diagnoses derived primarily from International Classification of Diseases (ICD) codes, which may limit diagnostic precision and preclude more detailed subphenotyping. Similarly, the ILAE dataset, while based on more detailed clinical phenotyping, still includes a substantial proportion of cases classified as unclassified, particularly within FE and GGE subtypes, which restricts the scope of subtype-specific analysis. Third, although we did not directly use UK Biobank epilepsy GWAS data in our analyses, the ILAE dataset includes UK Biobank participants for the “all epilepsy” phenotype. However, UK Biobank data were not used in the construction of the FE and GGE subtype GWAS in the ILAE study, thereby minimizing potential bias and type I error in the subtype-specific MR analyses. To further complement our findings and ensure independence from UK Biobank data, we also incorporated summary statistics from the FinnGen study, which does not overlap with UK Biobank. Fourth, despite the application of various filtering methods, some associations exhibited heterogeneity. This may reflect the complex genetic architecture of epilepsy and sleep-related traits and highlights the need for replication in larger, more precisely phenotyped cohorts using robust methods. In addition, all sleep-related traits in this study were derived from self-reported questionnaires, which may be subject to misclassification and reporting bias. Individual differences in the perception and reporting of subjective traits, such as chronotype or insomnia symptoms, may attenuate associations in GWAS and introduce bias toward the null in subsequent MR analyses. Future studies incorporating objective measures such as accelerometer-derived sleep metrics [29] may provide complementary insights and enhance the reliability of causal inference. Fifth, this study was based entirely on publicly available GWAS summary statistics and did not include individual-level clinical data. While this approach is widely adopted in MR studies and allows for efficient assessment of causal inference on a population scale, it limits our ability to validate genetic findings using direct phenotypic or clinical correlations. Although MR can provide insight into the potential causal effect of genetic liability for sleep-related traits on epilepsy (or vice versa), real-world clinical expression of these traits may be influenced by various non-genetic factors such as comorbidities, medications, environmental exposures, and lifestyle. Future studies incorporating deeply phenotyped patient cohorts, including EEG or sleep studies in people with epilepsy, would provide valuable complementary evidence and help confirm the clinical relevance of our findings. Sixth, we did not conduct sex-specific MR analyses due to the unavailability of sex-stratified GWAS summary statistics for most phenotypes. Considering the known sex differences in sleep patterns and epilepsy presentation, further studies with sex-disaggregated data are warranted to explore potential sex-specific causal relationships. Lastly, the datasets used in this analysis are restricted to individuals of European ancestry, which limits the generalizability of our findings to other populations.

## 5. Conclusions

Our study supports a subtype-specific and bidirectional causal relationship between epilepsy and sleep-related traits. While some of these findings are consistent with previous research, several of the observed causal relationships identified through MR warrant further clinical investigation to clarify potential bidirectional effects. These results may inform future studies aimed at identifying mediating mechanisms linking epilepsy and sleep traits, and at exploring whether specific epilepsy subtypes or sleep-related disturbances serve as intermediaries in pathways to other clinical outcomes. As both epilepsy and sleep traits are, to some extent, modifiable and clinically identifiable, targeted management of either may offer therapeutic benefits. Further research is needed to determine whether addressing one domain can mitigate downstream complications and improve overall patient outcomes.

## Figures and Tables

**Table 1:** Summary of GWAS datasets used in the present study.

Phenotype	Type of Trait	Sample Size (Case/Control or Increasing Ordinal Levels)	Author, Published Year	Consortium
Sleep duration	Continuous	446,118	Dashti H, et al., 2019 [29]	UK Biobank
Long sleep duration	Binary	411,934 (106,192/305,742)	Dashti H, et al., 2019 [29]	UK Biobank
Short sleep duration	Binary	339,926 (34,184/305,742)	Dashti H, et al., 2019 [29]	UK Biobank
Daytime sleepiness	Ordinal (4 levels)	452,071 (347,285/92,794/11,963/29)	Wang H, et al., 2019 [30]	UK Biobank
Daytime napping	Ordinal (3 levels)	452,633 (255,746/172,897/23,990)	Dashti H, et al., 2021 [31]	UK Biobank
Insomnia	Binary	453,379 (345,022/108,357)	Lane J, et al., 2019 [32]	UK Biobank
frequent insomnia	Binary	386,988 (109,548/277,440)	Watanabe K, et al., 2022 [33]	UK Biobank
Chronotype	Ordinal (5 levels)	449,732 (35,818/115,090/46,538/144,731/107,555)	Jones S, et al., 2019 [34]	UK Biobank
Morning Person	Binary	403,195 (252,287/150,908)	Jones S, et al., 2019 [34]	UK Biobank
Epilepsy cohort 1 (ILAE)	Binary	All epilepsies (European descent): 69,995 (27,559/42,436) Focal epilepsy (FE): 14,939 - FE with hippocampal sclerosis: 1260 - FE with other lesions: 4213 - Lesion-negative FE: 5778 Genetic generalized epilepsy: 6952 - Juvenile myoclonic epilepsy: 1732 - Childhood absence epilepsy: 1049 - Juvenile absence epilepsy: 662 - Generalized tonic–clonic seizures alone: 485	International League Against Epilepsy Consortium on Complex Epilepsies, 2023 [35]	International League Against Epilepsy Consortium on Complex Epilepsies
Epilepsy cohort 2 (FinnGen)	Binary	Epilepsy (G6_EPLEPSY): 390,250 (15,645/374,605) Focal epilepsy (FE): 493,978 (9275/484,703) Generalized epilepsy (GE): 486,387 (1690/484,697) Status epilepticus (G6_STATUSEPI): 375,973 (1368/374,605)	FinnGen Release: R12, 4 November 2024	FinnGen study

**Table 2:** Significant causal associations from epilepsy to sleep-related traits. This table summarizes significant MR results from epilepsy subtypes to sleep-related traits. For each exposure-outcome pair, the estimated effect size (β), 95% CI, unadjusted *p* value, and adjusted *p* value (for multiple comparisons) are reported. Results of heterogeneity tests (Cochran’s Q) and horizontal pleiotropy tests (Egger intercept) are also presented. Bold indicates significance in adjusted *p* values, heterogeneity tests, or pleiotropy tests. Pleiotropy test for FE with other lesions was not conducted due to the limited number of instrumental SNPs. * Became significant (*p* = 0.042) when multiple testing correction was limited to epilepsy subtypes, excluding all epilepsy, FE, and GGE. Abbreviations: MR, Mendelian randomization; CI, confidence interval; FE, focal epilepsy; SNP, single-nucleotide polymorphism; GGE, genetic generalized epilepsy; *p*_adj, adjusted *p* value for multiple comparisons; SE, standard error; ILAE, International League Against Epilepsy Consortium on Complex Epilepsies; JME, juvenile, myoclonic, epilepsy; JAE, juvenile absence epilepsy; HS, hippocampal sclerosis; GTCSA, generalized tonic–clonic seizures alone.

							Pleiotropy			Heterogeneity		
Cohort	Exposure	Outcome	SNPs	β (95% CI)	*p*	*p*_adj	Egger Intercept	SE	*p*	Cochran’s *Q*	*I* ^2^	*p*
ILAE	JME	Sleep duration	35	−0.016 (−0.026, −0.005)	0.004	**0.040**	−0.003	0.002	0.270	50.76	34	**0.032**
	JME	Long sleep duration	36	−0.003 (−0.006, −0.000)	0.022	0.219	−7.31 × 10^−5^	0.001	0.907	37.13	35	0.371
	FE with other lesions	Short sleep duration	2	−0.037 (−0.062, −0.011)	0.005	**0.048**				0.01	1	0.912
	All epilepsy	Daytime sleepiness	34	−0.018 (−0.030, −0.005)	0.007	0.065	−0.002	0.001	0.064	34.76	33	0.384
	GGE	Daytime sleepiness	54	−0.008 (−0.016, −0.001)	0.037	0.122	−0.002	0.001	0.115	104.26	53	**<0.001**
	FE	Daytime sleepiness	25	−0.018 (−0.035, −0.001)	0.037	0.122	−0.001	0.002	0.522	44.92	24	**0.006**
	JAE	Daytime napping	7	0.011 (0.003, 0.019)	0.009	0.088	0.003	0.009	0.732	8.91	6	0.179
	FE with HS	Insomnia	12	0.010 (0.003, 0.017)	0.006	0.060 *	0.000	0.001	0.751	11.91	11	0.371
	GTCSA	Insomnia	3	−0.011 (−0.021, −0.001)	0.025	0.124	0.009	0.009	0.492	1.30	2	0.521
FinnGen	Epilepsy	Daytime sleepiness	22	0.014 (0.005, 0.023)	0.002	**0.001**	−0.001	0.001	0.264	31.03	21	0.073
	Epilepsy	Insomnia	22	0.017 (0.006, 0.029)	0.004	**0.014**	−0.001	0.001	0.500	28.00	21	0.140

**Table 3:** Significant causal associations from sleep-related traits to epilepsy. This table summarizes significant MR results from sleep-related traits to epilepsy subtypes. For each exposure–outcome pair, the estimated effect size (β), 95% CI, unadjusted *p* value, and adjusted *p* value (for multiple comparisons) are reported. Results of heterogeneity tests (Cochran’s Q) and horizontal pleiotropy tests (Egger intercept) are also presented. Bold indicates significance in adjusted *p*-values, heterogeneity tests, or pleiotropy tests. Abbreviations: MR, Mendelian randomization; CI, confidence interval; SNP, single-nucleotide polymorphism; *p*_adj, adjusted *p* value for multiple comparisons; SE, standard error; ILAE, International League Against Epilepsy Consortium on Complex Epilepsies; JME, juvenile myoclonic epilepsy.

							Pleiotropy			Heterogeneity		
Cohort	Exposure	Outcome	SNPs	β (95% CI)	*p*	*p*_adj	Egger Intercept	SE	*p*	Cochran’s *Q*	*I* ^2^	*p*
ILAE	Long sleep duration	JME	3	5.560 (1.190, 9.931)	0.013	0.114	−0.070	0.064	0.474	1.78	2	0.411
	Chronotype	JME	102	−0.207 (−0.398, −0.016)	0.034	0.152	0.006	0.007	0.395	111.02	101	0.233
FinnGen	Daytime sleepiness	Epilepsy	33	0.820 (0.208, 1.433)	0.009	**0.039**	0.002	0.010	0.840	26.97	32	0.719
	Daytime napping	Epilepsy	79	0.432 (0.114, 0.751)	0.008	**0.039**	−0.001	0.006	0.919	83.02	78	0.328
	Frequent insomnia symptoms	Epilepsy	11	0.217 (0.018, 0.416)	0.033	0.801	−0.007	0.011	0.522	7.50	10	0.677
	Daytime napping	Status epilepticus	79	1.430 (0.408, 2.453)	0.006	**0.028**	−0.013	0.018	0.459	71.83	78	0.675
	Frequent insomnia symptoms	Status epilepticus	11	0.996 (0.336, 1.657)	0.003	**0.028**	0.006	0.036	0.865	8.16	10	0.613

## Data Availability

Data is contained within the article or Supplementary Material.

## Supplementary Materials

The following supporting information can be downloaded at: https://www.mdpi.com/article/10.3390/brainsci15070749/s1, Table S1: Comparison of previous Mendelian randomization studies on sleep, epilepsy, and related traits. Table S2: Full bidirectional Mendelian randomization results between epilepsy and sleep-related traits across the ILAE and FinnGen cohorts.

## Abbreviations

The following abbreviations are used in this manuscript:

EEG	Electroencephalography
MR	Mendelian Randomization
GWAS	Genome-Wide Association Study
IV	Instrumental Variable
ILAE	International League Against Epilepsy
FE	Focal Epilepsy
HS	Hippocampal Sclerosis
GGE	Genetic Generalized Epilepsy
JME	Juvenile Myoclonic Epilepsy
CAE	Childhood Absence Epilepsy
JAE	Juvenile Absence Epilepsy
GTCSA	Generalized Tonic-Clonic Seizures Alone
SE	Standard Error
CI	Confidence Interval
SNP	Single Nucleotide Polymorphism
LD	Linkage Disequilibrium
IVW	Inverse Variance Weighted
ESES	Encephalopathy related to Status Epilepticus during Sleep
CSWS	Continuous Spike and Wave during Sleep
ICD	International Classification of Diseases
