# Supplementary material for: Causal Relationship Between Epilepsy, Status Epilepticus and Sleep-Related Traits: A Bidirectional Mendelian Randomization Study
Source: Brain Sci. 2025 Jul 14;15(7):749. doi: 10.3390/brainsci15070749 (PMC12294028; doi:10.3390/brainsci15070749)
Supplement: Supplementary file 1 [file brainsci-15-00749-s001.zip › Supplementary Material_code.pdf]

```
library(TwoSampleMR)
library(MRPRESSO)

# Step 1: Format exposure and outcome data for MR analysis
# (Assume exposure_data and outcome_data have been pre-loaded from GWAS summary statistics)

exp_dat<-format_data(exposure_data, type = "exposure")
out_dat<-format_data(outcome_data, type = "outcome")


# Step 2: Select SNPs reaching genome-wide significance (or a relaxed threshold)

pval_threshold<-5e-08
exp_dat <- exp_dat %>% filter(pval.exposure < pval_threshold)


# Step 3: Perform LD clumping using 1000 Genomes EUR panel

exp_dat<-exp_dat %>%
  clump_data(.,
             clump_kb = 10000,
             clump_r2 = 0.001,
             clump_p1 = 1,
             clump_p2 = 1,
             pop = "EUR",
             bfile = "/path/to/1000G_EUR_Phase3", # reference panel
             plink_bin = genetics.binaRies::get_plink_binary()
)


# Step 4: Harmonize data

harm_out <- harmonise_data(
  exposure_dat = exp_dat,
  outcome_dat = out_dat,
  action = 3)


# Step 5: Filter SNPs with a minor allele frequency < 0.01, F statistics ≤ 10, or significantly associated with
both the exposure and outcome

maf_threshold <-0.01
harm_out <- harm_out %>%
  dplyr::filter(eaf.exposure >= maf_threshold) %>%
  dplyr::mutate(F_stat = (beta.exposure^2) / (se.exposure^2)) %>%
  dplyr::filter(F_stat > 10) %>%
  filter(pval.exposure < pval_threshold & pval.outcome > pval_threshold)


# Step 6: Additional SNP filtering based on confounders, influence, and pleiotropy
# See manuscript for details on confounder screening and pleiotropy filtering


# Step 7: Perform MR analysis

mr_out<-mr(harm_out)
```

```r
# List of sensitivity analyses

# (a) Leave-one-out analysis
loo_results <- mr_leaveoneout(harm_out)
mr_leaveoneout_plot(loo_results)

# (b) MR-PRESSO
mr_presso_results <- mr_presso(
  BetaOutcome = "beta.outcome",
  BetaExposure = "beta.exposure",
  SdOutcome = "se.outcome",
  SdExposure = "se.exposure",
  OUTLIERtest = TRUE,
  DISTORTIONtest = TRUE,
  data = harm_out,
  NbDistribution = 1000,
  SignifThreshold = 0.05
)

# (c) Heterogeneity (Cochran's Q and I2)
heterogeneity_results <- mr_heterogeneity(harm_out)
Q <- heterogeneity_results$Q[heterogeneity_results$method == "MR Egger"]
Q_df <- heterogeneity_results$Q_df[heterogeneity_results$method == "MR Egger"]
I2 = max(0, (Q - Q_df) / Q) * 100

# (d) Horizontal pleiotropy (Egger intercept)
pleiotropy_results <- mr_pleiotropy_test(harm_out)
```
